# Structures and objectives of day hospices – Design of a logic model

**DOI:** 10.1186/s12904-026-02356-x

**Published:** 2026-10-09

**Authors:** Ulrich Kaiser, Ursula Vehling-Kaiser, Felix Kaiser, Moritz Fiedler, Michael Rechenmacher, Elèene Kaiser, Florian Kaiser

**Affiliations:** 1 Clinic and Polyclinic for Internal Medicine III, University Hospital Regensburg, Franz-Josef-Strauß-Allee 11, Regensburg, 93053 Germany; 2 Day Hospice Adiuvantes, Vilsbiburg, Germany; 3 VK&K Studien GbR, Landshut, Germany; 4 Center for Palliative Medicine, University Hospital Regensburg, Regensburg, Germany

**Keywords:** Day hospice, Palliative care, Program theory, Logic model, Qualitative research

## Abstract

**Background:**

Day hospices represent a new component of outpatient palliative care in Germany. They focus on improving the quality of life and symptom control for patients with advanced, incurable diseases and aim to relieve the burden on family caregivers. However, a uniform theoretical framework for day hospices has not yet been established.

**Methods:**

To develop a logic model for day hospices, data were obtained from a retrospective literature review and qualitative, semi-structured telephone interviews with 11 patients and 5 relatives. The logic model was developed by the author team using a nominal group process and subsequently cross-checked by five patients and two staff members of a day hospice.

**Results:**

The final logic model comprised 18 categories and 105 program elements, organized into five pillars: resources, activities, output, outcome, and impact. It describes the functioning of a day hospice and its expected short- and long-term effects logically and coherently, based on key organizational, structural, and personnel requirements, as well as the services and interventions based on these requirements.

**Conclusions:**

The developed logic model provides an initial program theory for day hospices. It offers a clear and structured framework for understanding their functions and expected outcomes. The model can serve as a practical foundation for establishing new day hospices and for guiding future research and quality development in this emerging area of palliative care.

**Trial registration:**

The study was registered as part of the IMPULS-Study in the German Register of Clinical Studies (DRKS-ID: DRKS00031613; Date of registration: 04 April 2023) and the Display portal of the Center for Clinical Studies of the University Hospital Regensburg (Z-2022-1734-6; Date of registration: 01 October 2022).

**Supplementary Information:**

The online version contains supplementary material available at https://doi.org/10.1186/s12904-026-02356-x.

## Background

Day hospices represent a relatively new, specialized and little-researched form of outpatient palliative care. Essentially, they aim to enhance “the independence and (.) quality of life of individuals with a life-limiting illness, primarily through social engagement (e.g., group and artistic activities, café), rehabilitative measures, physical therapy, occupational and artistic therapies (e.g., art and music therapy), and/or medical care for symptom control” [1]. An additional benefit is the relief provided to family caregivers by offering palliative patients structured daily or hourly care, which can help stabilize the family environment and support continued care at home [2–5].

Previous studies have shown high levels of patient satisfaction with day hospice care [2, 6], although findings on improvements in quality of life remain inconsistent [7, 8]. These discrepancies may reflect methodological variation and structural heterogeneity among facilities studied [1]. Nevertheless, day hospices are expected to yield benefits for the German healthcare system [9].

Day hospices serve patients with advanced and incurable diseases. They operate on weekdays and during core working hours, after which patients return home [9]. High quality palliative care is provided by specialized palliative care nurses, while the involvement of other professional groups remains optional in Germany [9, 10]. The organizational, structural, and personnel models of day hospices vary nationally and internationally [1], despite emerging country-specific recommendations [11, 12]. Due to differences in healthcare systems, international models cannot be directly applied to the German context [1, 13]. The German S3 guideline distinguishes between medical models, social models, or combinations of both, depending on the approach taken [1]. It is often difficult to draw a clear distinction between the various care models. Depending on their focus, there may also be overlaps with palliative day care clinics [1, 4]. Initial theoretical structural recommendations for German day hospices have been proposed [14], but, their implementation remains limited: day hospices currently exist only sporadically in Germany [15–17] and there is a lack of a nationwide framework for their systematic integration into the medical care landscape [16]. Consequently, data and experience regarding the structure and short- and long-term effects of day hospices in Germany are still scarce.

The IMPULS study is a quality-assured investigation of day hospices based on the Medical Research Council Framework for the Development and Evaluation of Complex Interventions [18] (DRKS-ID DRKS00031613, Z-2022-1734-6 [19]), . It aims, among other things, to generate robust real-world data on palliative medical and nursing care in day hospices. The previous lack of standardization among day hospices has made it difficult to conduct a detailed study of their effects. Therefore, in its initial phase, the study seeks to systematically develop a uniform program theory of day hospices that describes the structural and intervention-related framework as well as the expected short- to long-term effects. For this purpose, a theoretical model (logic model) was established that provides a comprehensible, comparable, and reliable foundation for future research and practice. The present work describes a newly developed logic model for day hospices that explains how day hospices function as a whole.

## Methods

### Ethics

This study was conducted as part of the IMPULS study (investigation into the implementation, realization, and benefits of a day hospice, German Clinical Trials Register: DRKS-ID DRKS00031613, Center for Clinical Trials of the University Hospital Regensburg: Z-2022-1734-6), which was approved by the Ethics Committee of the University of Regensburg (Approval No. 22-3012_1-101). All approvals were granted in full compliance with the principles of the Declaration of Helsinki.

### Development of the logic model

To ensure a clear and methodologically sound development process, the structured recommendations of the Kellogg Foundation for logic model development were followed [20]. The necessary basic information for the development process was gathered through a systematic analysis of the situation and needs for day hospices in two key stages: previously published national/international literature on day hospices and newly conducted qualitative interviews regarding the views and wishes of affected patients/guests and their relatives [20]. On this basis and based on their clinical experience, the authors developed an initial draft of the logic model through a nominal group process. The aim was to systematically illustrate the links between the resources deployed, the planned activities and the desired outcomes. In order to involve key stakeholders in the development process, the draft was subsequently cross-checked by staff and guests of the day hospice. On this basis, the logic model was finalized by the authors.

### Describing the logic model

**Fig. 1:**
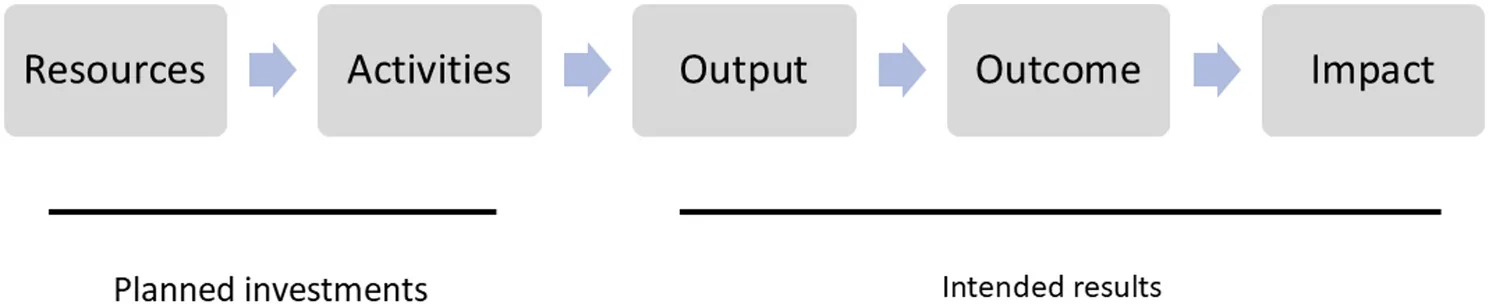
The logic model [21]

The logic model was used as the conceptual framework for developing the program theory of day hospices [21, 22]. This model provides a theoretical and logically structured foundation that explains under what conditions and through which mechanisms specific short-, medium-, and long-term effects or impacts can be expected. It is based on a logical sequence of events following the “if-then” principle, illustrating the underlying logic of impact and how a program or intervention functions as a whole. The fundamental objective of the logic model is to present a program in a clear and accessible manner, enabling visualization of how it works and the results it produces. It should also help to identify and precisely define research questions or focal aspects within complex interventions. The logic model [21] is based on five stages, divided into two sub-areas (Fig. 1):

#### Planned investments (resources required to implement the program)

1. Resources (framework conditions): e.g., necessary human, financial, and organizational resources.
2. Activities (actions to achieve aims): Describes how the program utilizes the required/available resources to achieve the intended program changes and program outcomes.

#### Intended results (desired outcomes of the program)

3. Output: Immediate results/effects of program activities.
4. Outcome: Short- to medium-term effects of program activities.
5. Impact: Long-term effects of program activities, potentially extending to broader systemic or societal context.

### Data collection

To create an evidence-based data foundation for developing the logic model and its individual components, two preparatory steps were undertaken:

1. Literature search: The search strategy was based on the Systematic Approaches to a Successful Literature Review [23]: PubMed, Google Scholar, and the Cochrane Library were used as databases. Search terms included “day hospice,” “structure,” “implementation,” “organization,” “results,” and “planning.” The search period covered publications from 1990 to 2025. Only publications that explicitly described the services, structures and effects of day hospices were included. Studies lacking these descriptions were excluded. As a recent systematic review on day hospices already exists [13], our literature review was not designed as a comprehensive systematic review. Consequently, no quality assessment was carried out. To ensure a high standard of evidence, particular emphasis was placed on current national and international recommendations, guidelines, and review articles. The main objective was to identify the existing structures, services and objectives of day hospices. The search results formed a basis for further analysis, evaluation and development of a logic model as part of the nominal group process described below.
2. Qualitative guided interviews: In collaboration with Sinus Markt- und Sozialforschung GmbH (Heidelberg), 16 qualitative semi-structured telephone interviews were conducted between February and June 2024, comprising 11 day hospice guests and 5 relatives of a single day hospice (average duration: 40–44 min) [24]. Telephone interviews were chosen to maximize scheduling flexibility and feasibility in the sensitive palliative care context. Guidelines ensure that all relevant aspects were addressed during the interviews. The interview format enabled participants to express themselves openly and allowed the interviewer to explore relevant themes through follow-up questions. Interview guides were jointly developed by Sinus GmbH and the authors (UK, FK, and UVK) for both day hospice guests and relatives (see Appendix Tables 1 and 2). Each guide consisted of 5 main questions and 22 sub-points, structured around the five pillars of the logic model [21]. Topics focused on the perceived significance and impact of day hospice services, as well as the role of structural and programmatic elements in achieving the intended objectives. Written informed consent was obtained from all participants prior to their participation in the study, in accordance with ethical standards. All interviews were audio-recorded and transcribed anonymously. The analysis was based on the principles of qualitative empirical social research using hermeneutic text interpretation [25]. This is a qualitative analytical method for the interpretative analysis of interviews and their contextual meanings. It is based on the assumption that texts can only be understood by taking into account their linguistic, historical and social context. The analysis is carried out iteratively in the sense of the hermeneutic circle, in which individual passages of text are continuously related to the overall context. The aim is to systematically identify implicit meanings, underlying assumptions and central structures of meaning. The findings formed a second basis for the nominal group process described below.

### Development process

The authors UVK, UK, FK, MF, and MR (all with many years of experience in palliative care, day hospices or palliative care quality management) developed an initial draft of a logic model for day hospices using an nominal group process [26]: the nominal group technique is a structured method for systematic brainstorming and prioritisation within groups. Its aim is to capture as many perspectives as possible on an equal footing and to facilitate consensus-based decision-making. The process comprises several consecutive steps: first, based on the above data (literature/interviews) and their clinical experience the authors developed ideas to identify the key structural and functional elements of a day hospice individually and in writing. The following key themes were defined for the selection of elements: potential services, content and organisational/structural/staffing arrangements for day hospices; networking; potential effects of these measures; the objectives of day hospices; funding; structural integration into the healthcare system. The contributions were then collected in a facilitated joint meeting and documented visibly, without being evaluated immediately. This was followed by a joint discussion to clarify and refine the content. Within this framework, the findings from the existing literature were also discussed and selected on the basis of their relevance, methodological quality and potential biases [23]. Finally, the authors prioritized the proposals using a structured voting procedure. By combining individual reflection with group-based consensus-building, the method enables transparent and accountable decision-making [26].

In detail, the information identified was categorized and mapped to the key elements of the logic model:

- Compilation and classification of the authors’ individual findings according to the pillars of the logic model (Resources, Activities, Output, Outcome, Impact);
- Grouping of findings within each pillar into separate categories;
- Development of the initial version of the logic model.

This development process involves several steps: first, the initial situation and the problem to be solved were defined. Second, the available resources were identified. Building on this, the planned measures and activities were described. These lead to specific outputs or direct results (outputs), from which medium- and long-term effects (outcome and impact) were derived [20]. This approach enables a theory-driven and transparent development of the model and ensures that the assumed causal relationships are empirically and contextually grounded [20].

As the grouping, prioritisation and refinement of the individual elements for the logic model took place as a dynamic process within the framework of the nominal group process, the individual experiences and insights of the participating authors (as experts in the field of palliative care) were decisive in this process, in addition to the structural guidelines described above.

### Cross-checking and finalization

To assess the model’s practical applicability and everyday relevance, the initial draft was presented to two staff members and five guests of the day hospice, representing the primary target groups. They were given the opportunity to submit written comments and remarks. The aim of this iterative process was to assess the identified aims, resources, activities and assumed causal relationships for plausibility and practical relevance. This participatory revision process is in line with the Kellogg Foundation’s recommendations for developing theory-driven and context-specific logical models [20]. The logic model was finalised by the team of authors following the evaluation and incorporation of stakeholder feedback. In doing so, the identified relationships between the individual pillars of the logic model were consolidated, checked for their internal and interdependent logic, and transformed into a consistent structure, resulting in a comprehensible and theory-based model that fully and plausibly reflects the underlying impact logic of a day hospice [20].

## Results

### Results of literature search

A total of 42 publications addressing the implementation or operation of day hospices were included in the analysis. Among these, four key publications (an S3 guideline, an international recommendation for action, a national recommendation for action, and especially a review by Terjung et al. [1, 11, 13, 14]) were identified as particularly relevant due to their close alignment with the German healthcare system, their topicality, and/or their broad overview of the current literature. Table 1 summarizes the aspects identified as essential and relevant for day hospices.

**Table 1:** Key structural and organizational aspects of day hospices identified through the literature analysis

Key aspects	Key categories and program elements (in parentheses)
*Definition*	• Core tasks of the day hospice • Definition of day hospice (S3 guideline, social/medical/combined approach)
*Structural aspects*	• Location (close to home) • Arrival (transport connections, ensuring arrival and departure) • Operating hours (5 days, full-day/half-day programs) • Cooperation/networks (integration into local care structures) • Spatial facilities (rest rooms, common rooms, protected/family atmosphere) • Staffing (palliative care nurses as core team, multidisciplinary team) • Optional occupational groups • Admission criteria • Admission process (admission capacities, occupancy planning, scheduling) • Discharge process • Dealing with appointment cancellations • Emergency planning/emergency equipment, advanced care planning • Catering
*Content aspects*	• Offers/measures/activities for patients (animal/art/music therapy, physical/occupational/aromatherapy, psychotherapy, wound management, symptom control/monitoring, medical consultation, community activities (baking, handicrafts, shared meals/excursions, board games), social and legal counseling, wellness (baths, massages, hairdressing, pedicures), spirituality/devotion, introduction to palliative care/end-of-life care) • Services for relatives (counseling/information, guidance, e.g., aids/care measures) • Daily structure/program • Scope of medical care • Scope of nursing care • Scope of psychosocial care
*Public relations*	• Increasing awareness (digital/print media, taster days, word of mouth) • Referrers/collaborating partners (general practitioners and specialists, SAPV, hospice association, self-help groups) • Allocation criteria/reasons
*Funding*	• Securing financing • Funding amount • Cost-effectiveness (conservation of hospital resources, reduction in hospital stays)
*Goals to be achieved*	• Assessment/performance measurement tools • Objectives for patients (remaining in their home environment for longer, symptom management, coping with illness, gaining autonomy, reducing depression/loneliness, improving quality of life/self-esteem/dignity, social integration, promoting personal resources) • Objectives for relatives (reducing family stress, providing relief, coming to terms with the overall palliative situation/end of life of the patient, reducing pathological grief/depression, reducing financial burden, improving quality of life)

### Results of qualitative interviews

Overall, the assessments of patients and relatives were largely consistent, although some differences emerged in the relative importance assigned to specific aspects.

#### Spatial facilities

For both patients and their relatives, the common room represents the heart of the day hospice and fulfills core needs for interaction, social contact, and shared experiences. This is supported by communal meals and group activities. Integrated reclining chairs provide areas for rest and individual space while allowing patients to remain socially connected.

Single rooms were emphasized as important spaces for retreat and rest by patients and (even more so) by their relatives. These rooms meet the desire for greater individuality and allow palliative patients with reduced physical capacity or temporarily increased need for rest to participate in day hospice care. They can also serve multifunctional purposes, such as for therapeutic applications or individualized sessions. Where such rooms are unavailable, an additional space for supportive services (e.g., physical therapy or aromatherapy) was considered desirable.

Quote: “There are also a few quiet rooms for people who are particularly distressed, (…) who (…) simply want to be alone in a room and have some peace and quiet. That’s really (…) important.” (09)

Regarding other facilities, participants highlighted the importance of an on-site kitchen, either integrated within or separate from the main area. For sanitary facilities, including at least toilets and, if applicable, showers, accessibility and adequacy relative to the number of guests were key priorities. When medical services are offered, a dedicated physician’s office is considered desirable to ensure confidential and sensitive care.

An outdoor area was also seen as highly valuable. It contributes to well-being, offering opportunities for social interaction and short walks adapted to guests’ physical abilities. Accessibility, comfortable seating, and an aesthetically appealing environment were considered essential.

Quote: “And especially outside (…). I think that’s good. (…) I mean, if I were locked up like that, it would hurt me not to be able to go outside all day.” (02)

#### Medical, social, and supportive services

When offered in day hospices, on-site physician care is highly valued by both patients and even more by their relatives, contributing significantly to their sense of safety and comprehensive care. Minor medical examinations (such as blood sampling, pulse measurement, or wound care) are regarded as particularly beneficial, as they reduce the need for time-consuming external visits to physicians. Psychotherapeutic care and the option of parenteral nutrition were also perceived as very helpful services.

Quote: “It’s easy, I don’t have to drive long distances to get my blood taken or to see my family doctor anymore. Everything is done at the hospice.” (04)

Social services were considered an indispensable component of day hospice care by both patients and relatives. A broad and diverse range of social activities was described as essential to the sense of “all-round care” and to maintaining a fulfilling daily routine. Reported activities included board games, handicrafts, arts and crafts, lectures, discussions, church services, celebrations, and creative activities. Such activities were viewed as crucial for promoting mental and physical well-being, fostering a renewed sense of normalcy, emotional support, and self-perception beyond the illness.

Quote: “There’s pottery making. There’s wreath making, Advent (…), there’s really everything on offer. You’re really well looked after in every way. (…).” (04)

Cosmetic treatments such as hairdressing, nail care, and pedicures were perceived as particularly positive and practical offerings that also help alleviate the burden on relatives. Many patients indicated that, without the day hospice, they would be unable or would find it difficult to access these services due to physical, organizational, or financial constraints.

Quote: “You get offers for things (e.g., haircuts, manicures, pedicures) that are difficult to fit into your everyday life, but that you can easily incorporate into your stage performance.” (01)

Other activities receiving consistently positive reviews include physiotherapy, which helps prevent increasing immobility, and group excursions organized by the day hospice. Although the latter are suitable mainly for more mobile guests, such outings were described as valuable opportunities to experience a change of environment and strengthen the sense of community. Participation in aromatherapy and animal therapy was also viewed as mood-enhancing, calming, and beneficial for emotional well-being. Patients generally regarded social law counseling and support services as helpful, though they reported a lower perceived need for these offerings compared to their relatives.

All available programs were explicitly welcomed, particularly due to the emphasis on voluntary participation, individual choice, and the freedom to withdraw at any time.

Quote: “(…) well, I’m pleasantly surprised, yes. And above all that you do have a certain amount of freedom there.” (02)

#### Human resources

Both patients and their relatives identified the presence and quality of nursing staff as the most important factor for successful care in a day hospice. Attentive, individualized, and compassionate care, while maintaining appropriate professional boundaries, was regarded as essential. Equally important was the staff’s role as a point of contact and source of support for relatives.

Quote: “The staff is exceptional. (…) from the very first moment, you are taken care of. You are looked after. And I have to say that was actually my last resort (…).” (04)

Participants emphasized the need for continuous and consistent care provided by motivated and empathetic nursing personnel, ideally with additional specialist palliative care training. Such staffing was considered a fundamental prerequisite in a day hospice for maintaining quality and continuity of care.

Among other professional groups involved, regular weekly visits by a (general)physician were described as particularly valuable. However, the permanent presence of medical specialists was viewed as neither necessary nor feasible and was considered beyond the intended scope of the day hospice concept. Nonetheless, participants stressed the importance of having a plan for emergencies. Additional professionals, such as hospice companions, nutritionists, or pastoral caregivers, were also valued, though not regarded as an indispensable component (*conditio sine qua non*) of day hospice care.

#### Financial resources

For most patients, the financing of day hospices is an unknown quantity beyond nursing care/health insurance payments and remains largely unfamiliar. Given the overall cost, self-financing of the entire service by users is generally not feasible. However, both patients and their relatives indicated that individual financial contributions, scaled to personal financial capacity, would be possible. When discussing potential funding models, both groups emphasized the importance of government subsidies and donations in the spirit of solidarity.

Quote: “… and I would also support government assistance.” (07)

#### Organizational resources

A stress-free, reliable, and clearly organized journey to the day hospice was considered essential. The ideal travel distance was described as no more than ten kilometers or lasting approximately ten minutes. Nevertheless, participants acknowledged that longer travel times may be acceptable given the high perceived value of day hospice services. Taxis (typically approved by health insurance) were the most commonly used means of transportation, followed by rides provided by relatives. Public transportation and independent travel were rarely used due to the health condition of the patients.

Operating hours on weekdays from 8 a.m. to 4 p.m. were viewed as appropriate and convenient. Full-day attendance was preferred, as half-day visits were considered more of an exception due to the associated effort. Weekend services were generally not regarded as necessary.

Quote: “Yes that’s fine. (…) It runs from 8 a.m. to 4 p.m.” (09)

#### Social resources

The social engagement with issues surrounding dying and death perceived by the patients surveyed (to a greater extent than by their relatives) is low and is mostly suppressed or treated as taboo subjects. As a result, such matters tend to be made as invisible as possible. This also affects public visibility and perception of day hospices, leading to misinterpretations and negative associations.

Quote: “That was at first. Yes. Because you’re so stupid, because you’ve never dealt with it when you’re healthy. Then it’s just a hospice, and that’s a hospice for the dying. That’s just what’s in your head. That’s completely normal. Well, for me it was completely normal.” (04)

Despite these perceptions, or perhaps precisely because of them, participants considered day hospices to be an important institution in an aging society, both for relieving the burden on family caregivers and for helping patients remain connected to their everyday lives. The opportunity to benefit from the supportive environment of a day hospice while gradually coming to terms with their condition was regarded as particularly valuable.

Quote: “It’s a kind of stopover. And that would make it easier (…) for many elderly people to slowly say goodbye to their homes. Yes, at some point the time will come (…). No one stays here.” (09)

Patients and relatives also agreed that day hospices can play an important role in increasing public awareness and acceptance of illness, dying, and death. By providing visible community-based models of care, they may help to reduce the taboo surrounding these topics, particularly among younger generations who often lack exposure or education in this area.

Quote: “I think it’s more of a generational problem. (…) And I always think that there should be a little more education about the problem from an early age, maybe even in school (…)” (08)

#### Public relations

From the perspective of patients and their relatives, day hospices remain largely unknown to the general public and even to many individuals who might benefit from them, apart from a few well-publicized flagship projects. Broader public coverage and outreach efforts appear to be lacking. At present, referrals to day hospices occur mainly through a few cooperation partners. Patients and relatives expressed that broader referral pathways would be desirable, particularly through physicians, care centers, hospitals, and health insurance providers. Both groups emphasized the need for increased awareness-raising and media coverage (press, radio, television, digital media) or even word-of-mouth recommendations in order to raise the profile of day hospices and improve access for potential users.

Quote: “I didn’t know that such a thing existed (…) and since then I’ve been there too.”(07)

#### Objectives and effects of day hospices

The purpose of a day hospice is perceived as providing community, individualized medical and psychosocial care, and uplifting personal attention, which together have an immediate positive effect on mental and physical well-being, foster self-fulfillment, and help to counteract loneliness.

Quote: “That you are torn away from my everyday life, from being alone, that you are in society (…).” (07)

Quote: “(…) i feel like a new person when I come out of here (…). And then I have strength again.” (04)

Day hospices also provide temporary emotional relief for caregiving relatives, offering them time for themselves and helping to reduce feelings of strain and being overwhelmed.

Quote: “My daughter (…) has really spent a lot of time with me, and she has also been relieved a little.” (03)

From the medium-term perspective, patients view day hospice care as a means of regaining autonomy and maintaining independence. At the same time, both patients and relatives perceive it as a guarantee of continuity of care in the event of declining health, helping to reduce anxiety about future transitions, including admission to inpatient facilities (e.g., hospices).

In the long term, both groups associate the work of day hospices with broader societal effects, including greater social acceptance of serious illness and dying, increased volunteer engagement, increased willingness to donate, and a stronger social commitment to maintaining and financing such services. Furthermore, day hospices appear to reassure relatives about their own potential future care needs.

Quote: “It’s definitely a comforting thought if you ever find yourself in that situation.” (15)

### Results logic model

**Fig. 2:**
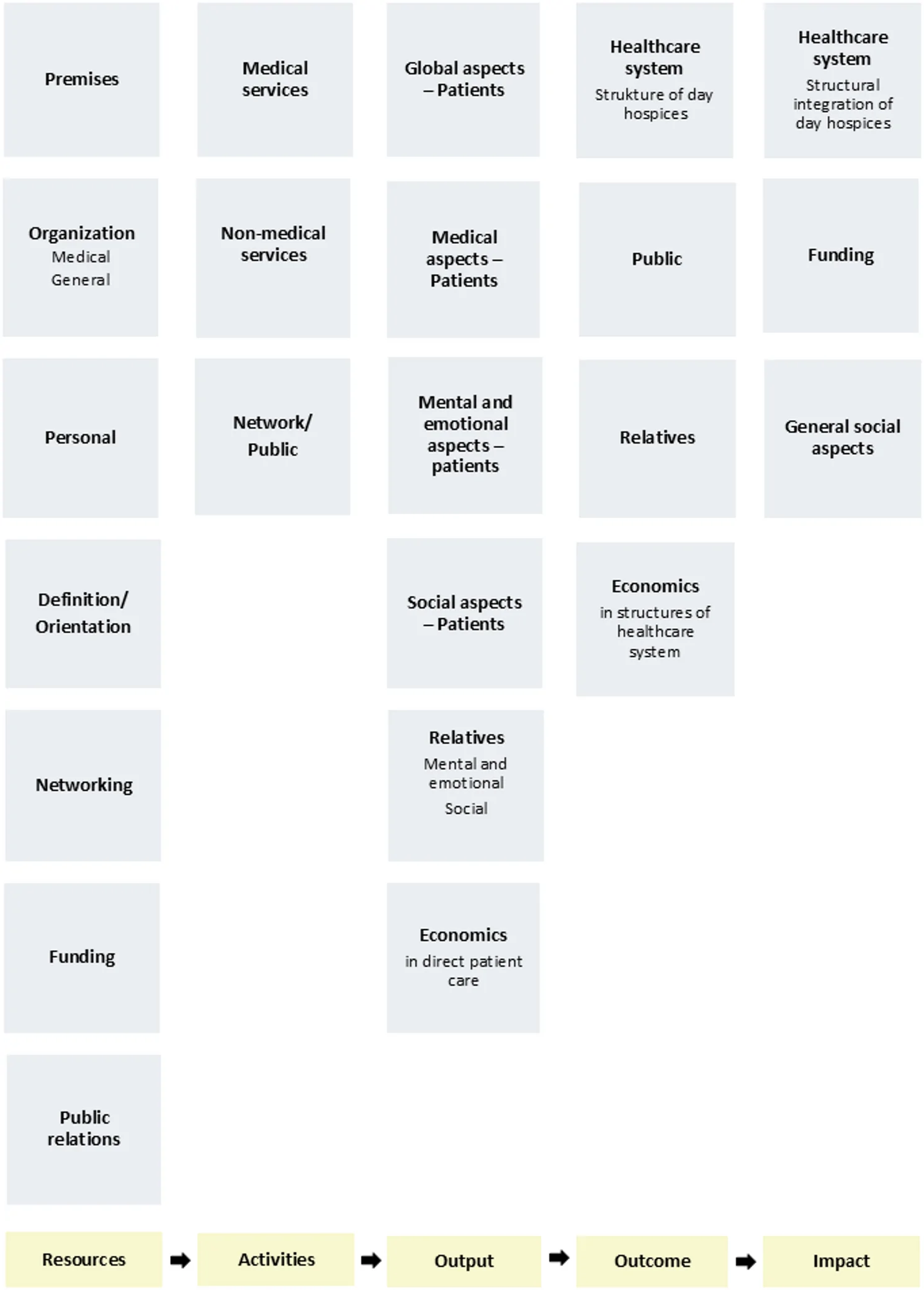
“Logic model of a day hospice”

A total of 18 categories containing 105 program elements were identified. During analysis, the program elements within each category were grouped thematically. The individual pillars of the logic model each comprise three to seven categories, with a total of 10–39 program elements (Fig. 2; Table 2). It is important to bear in mind that there may be overlaps between program elements, particularly in the categories of Output, Outcome and Impact. Individual elements, such as the integration of day hospices into outpatient care structures or the effects on the quality of life and stress levels of relatives, could therefore, in principle, fit into several categories.

#### Pillar 1–resources

This pillar describes the basic requirements for operating a day hospice. In addition to the essential physical and organizational resources, the securing of financial support, the integration of the day hospice into the local palliative care network, and effective public relations work were identified as essential foundations for successful operation. It also includes the fundamental requirements for implementing the interventions and services offered by the day hospice, such as personnel and collaborating partners.

#### Pillar 2–activities

Based on the resources described in pillar 1, this pillar outlines the specific services and operational activities required to achieve the objectives of a day hospice. These primarily include medical and nursing care as well as non-medical/social services for patients and, where applicable, their relatives. While the scope and nature of these services may vary depending on the organizational orientation of the facility, palliative medical and nursing care delivered by palliative care nurses is the central focus of day hospice operations.

#### Pillar 3–output

This pillar captures the immediate results of day hospice activities, which are expected to primarily affect patients and, secondarily, their relatives. Improvements and relief are expected at medical, social, and psychological-emotional levels. The initial effects on healthcare system structures may also begin to emerge at this stage.

#### Pillar 4–outcome

Given the limited life expectancy of patients, the medium-term effects are expected to be particularly important to relatives, especially in terms of reducing long-term psychological burden or trauma. Broader systemic effects may also become evident. In addition to economic benefits, these relate to the level of awareness and social acceptance of, and understanding for, day hospices, as well as the further development of health policy and the integration of day hospices into healthcare provision.

#### Pillar 5–impact

In the long term, the very positive findings suggest the potential to establish day hospices as a permanent, sustainably funded component of palliative care. A broader societal impact can also be anticipated, particularly through enhanced public attitudes toward serious illness, dying, and death, reflecting a more compassionate and open societal approach to end-of-life care.

**Table 2:** The detailed program elements of the logic model

Resources	Activities	Output	Outcome	Impact
**Premises** Location (convenient for transportation, close to home, quiet) Garden/Outdoor area Patient rooms/ individual rooms and relaxation rooms Areas of application Common rooms Physician's office, if applicable Kitchen, sanitary facilities, and bathrooms Staff rooms, storage rooms Possible spatial cooperation/synergy with other participants in hospice/ palliative care Accessibility	**Medical services** (Medical) and symptom-oriented treatment Palliative care Supportive treatment (physical therapy, occupational therapy, aromatherapy, psychotherapy, animal therapy, etc.) Appropriate and patient-oriented approach in emergencies Services for relatives (care instructions, counseling, etc.)	**Global aspects – Patients** Improvement in quality of life, mental and physical well-being Increased social integration/sense of community Maintaining/ promoting independence/ autonomy/self-fulfillment	**Healthcare system** Integration of day hospices into outpatient palliative care structures Elimination of interface problems Uniform structures and offerings Uniform definition for assessment tools/quality indicators	**Healthcare system** Comprehensive range of day hospices (urban and rural areas) Routine part of outpatient palliative care Reduction of bureaucracy
**Organization** Medical Advance Care Planning Emergency management Documentation (digital/analog) Quality management Further education/training General Opening hours Admission criteria Arrival and departure of patients Personalised appointment coordination Home economics Catering	**Non-medical services** Wellness Massages, manicures/pedicures, baths, hairdresser Art and music therapy Spiritual Care Consulting Palliative medicine Social law Psychosocial Community/leisure activities Group activities (board games, handicrafts, etc.) Shared meals Mutual exchange Adventure Therapy (group excursions, etc.) Places to retreat/rest	**Medical aspects – Patients** Improvement in symptom management Promotion of positive resources Introduction to palliative care/end of life	**Public** Increased awareness of day hospices Social acceptance of day hospices Reducing misinterpretations/fears about day hospices	**Funding** Ensuring that costs are covered (by health insurance companies) Financial independence of day hospices
**Personal** Palliative care nurses (core team) Multidisciplinary team including external specialists (physical therapist, pastoral care provider, nutritionist, (palliative) physician, etc.) Volunteer work Management/Administration Sufficient special qualifications	**Network/Public** Integration into local palliative care network Wide range of access options for patients through various referral sources (physicians, hospitals, care centers, etc.) Media relations	**Mental and emotional aspects – patients** Increased emotional well-being Preserving hope and patient dignity Reduction in depressive mood Support in dealing with dying and death/reducing anxiety	**Relatives** Mitigation of long-term consequences due to illness/death of patients Maintaining financial independence Increased security for relatives for their own future care needs	**General social aspects** Influence on how society deals with dying/death (making it visible, removing taboos, addressing the issue) Seriously ill patients become part of social life again Increased volunteer work Increased willingness to donate Society''s desire to establish and finance day hospices
**Definition/Orientation** Medical focus Social orientation Combined approach		**Social aspects – Patients** Staying at home for longer Creation of new social relationships Reducing social isolation Restoration of normality Experiencing uplifting appreciation	**Economics** Cost reduction Further expansion and ensuring of outpatient palliative care provision Relief for other care structures	
**Networking** Medical cooperation partners Social cooperation partners Demand planning		**Relatives** Mental and emotional Reduction of stress and family tensions Reduction of excessive demands Strengthening in dealing with dying and death/reducing fear Social Ensuring home care Relief in patient care/support Relief in everyday life		
**Funding** Framework agreement for day hospices Coverage by health insurance companies/donations Contact person at cost bearer		**Economics** Reduction in hospital stays Reduction of other supportive services Contribution to security of supply in an aging society		
**Public relations** Digital and print media Visiting days (relatives) Taster days (patients) Information about day hospices				

## Discussion

This work aimed to develop a program theory for day hospices based on a logic model. This was carried out as part of the IMPULS study and provides a theoretical foundation for further planned investigations [19]. At the same time, the model is intended to serve as a comparable, comprehensible, and easy-to-use framework for the structured establishment of future day hospices and for further research in this field.

Through its logical structure, the model describes and explains how a day hospice functions, beginning with the required resources, followed by the services and interventions, enableing, and culminating in the short-, medium-, and long-term effects. This systematic framework facilitates the precise formulation of research questions [27]. The model also supports the accurate classification of research questions within the overall context of the complex intervention represented by the day hospice.

By combining comprehensive retrospective data from previous studies with new qualitative data reflecting the perspectives of patients and relatives, the logic model provides a basis for understanding both the functioning and the potential scope and objectives of day hospices. The substantial overlap between the retrospective and qualitative findings reinforces the model’s credibility, while the complementary insights from each data source contribute to a more holistic understanding. The resulting logic model therefore serves as a reliable quality and planning tool for establishing new day hospices. Owing to its structured and sequential nature [21], the model supports systematic planning during the start-up phase and provides clear guidance throughout implementation. At the same time, it allows flexibility in the thematic and professional design of new day hospices, within the framework of applicable regional or national standards [1].

As day hospices represent a relatively new form of palliative care in Germany, only an initial set of specific quality indicators is available; however, these must be validated and continuously refined [28]. Such indicators are essential for ensuring high-quality medical and nursing care, enabling the systematic review of individual aspects of care and identification of opportunities for improvement [29, 30]. However, since the global parameter “quality of day hospice care” cannot be captured through a single global measure, but rather through a multidimensional assessment of partial aspects, an in-depth understanding of the mechanisms and structures of day hospices is necessary. The logic model presented here provides this understanding and can therefore make a meaningful contribution to the further development of a structured quality management system for day hospices, which remains an unmet need.

## Limitations

Several limitations should be considered when interpreting the study findings. In particular, the medium- to long-term effects of day hospices presented here are based primarily on the expectations and perspectives of patients, relatives, and experts, rather than on large-scale quantitative data. Given that day hospices represent a new form of care, particularly in Germany, such data are currently scarce and challenging to obtain. In particular, many of the proposed Outcomes and Impacts are therefore hypothetical effects rather than proven results. Future studies will be required to validate and, if necessary, adapt the logic model in light of empirical findings and practical experience. With respect to resources and activities, this model includes a broad range of basic requirements and measures/interventions that have already been successfully applied or are considered useful. However, the extent to which these elements can be realistically implemented in newly established day hospices remains questionable. It is also unclear whether all listed measures are indispensable for success, apart from those defined by contractual or regulatory frameworks. In practice, the selection and prioritization of interventions will likely need to be tailored to local conditions and needs. At present, it remains undetermined which specific components should be considered essential or prioritized for optimal outcomes in day hospice care. It is also important that the interviewees/interview data all come from or are associated with a single day hospice. Their experience of day hospices with different structures is therefore limited, and this may restrict their views and ideas. Consequently, parts of the logic model may reflect the structure and practices of this particular day hospice. It may therefore be necessary to evaluate or adapt the model for use in other day hospices. As the day hospice under investigation operates within the framework of the German healthcare system, adjustments may be required to take account of differences arising from other international healthcare systems. As there is no final distinction between day hospices and palliative day care clinics, our logic model for day hospices may overlap with the content and focus of palliative day care clinics [4, 31]. An exact distinction between these two forms of care was not part of the study objectives. Therefore, this point cannot be fully addressed here.

## Conclusions

The newly developed logic model provides an initial, context-informed and comprehensive theoretical framework for understanding and implementing day hospices. It encompasses all key components, from the required resources to the resulting services and interventions, and extends to the expected short-, medium-, and long-term effects. The model systematically explains the structure, processes, and theoretical foundations of day hospices clearly and coherently. This framework can serve as a practical foundation for future research, enabling studies to be planned with greater precision and focused on specific aspects of day hospice care. Furthermore, it can potentially facilitate the establishment of new day hospices in a more structured manner.

## Supplementary Information

Supplementary Material 1.

## Data Availability

All relevant data are within the paper. The raw data analyzed in this study are available upon reasoned request to the corresponding author.

## Abbreviations

DRKS: German Register of Clinical Studies
IMPULS: Investigation into the implementation, realization, and benefits of a day hospice
