# Supplementary material for: Structures and objectives of day hospices – Design of a logic model
Source: BMC Palliat Care. 2026 Oct 9;25:300. doi: 10.1186/s12904-026-02356-x (PMC13651420; doi:10.1186/s12904-026-02356-x)
Supplement: Supplementary file 1 [file 12904_2026_2356_MOESM1_ESM.docx]

**Appendix**

**Table 1** Interview guide for relatives

| **Question Number** | **Question** |
| --- | --- |
| **1.** | **Presentation** |
| 1.1. | To begin, could you tell me a little about yourself?  *(Prompt: Age, marital status, occupation, interests)* |
| 1.2. | Could you describe what a typical day looks like for you?  *(Prompt: What kinds of activities or responsibilities usually fill your day? How does caring for your relative fit into your daily routine?)* |
| 1.3. | Could you tell me a little about the person you care for as a family member?  *(Prompt: What is your relationship to them, and how would you describe their current situation or health condition?)* |
| **2.** | **Day hospice: perception and evaluation** |
| **2.1.** | **Significance and impact of a day hospice** |
| 2.1.1. | Today, I would like to talk with you about the idea of a “day hospice.” Could you describe what you believe is the main purpose and aim of a day hospice?  *(Prompt: What comes to mind when you think of a day hospice? What do you think it is meant to provide or achieve for patients and families?)* |
| 2.1.2. | What immediate or direct effects do you hope a day hospice can have, for you as a relative, for your family member, or in general? |
| 2.1.3. | Looking a bit further ahead (medium term), what effects or impact a day hospice might have in the next few years, for you as a relative, for the patient, or more broadly for the healthcare system or society? |
| 2.1.4. | What long-term effects (up to 10 years) do you think day hospices could have, for you as a relative, for the healthcare system, or for society as a whole? |
| **2.2.** | **Assessment of the importance of offers for achieving objectives** |
| 2.2.1. | In your personal view and experience, what services and facilities do you think are most important for a day hospice to offer in order to achieve the above-mentioned goals? |
| 2.2.2. | What kind of facilities or spaces do you think a day hospice needs to provide these services?  *(Prompt: For example, what are your thoughts on areas such as restrooms, activity rooms, or outdoor spaces like a garden or balcony?)* |
| 2.2.3. | In your opinion, which medical services are most important for achieving the goals of a day hospice?  *(Prompt: For example, services such as psychotherapy, wound care, pain management, nutritional support, etc.?)* |
| 2.2.4. | Which social services do you think are important for a day hospice?   - Cosmetic treatments (e.g., pedicures, hairdressing, etc.)? - Complementary measures (e.g., aromatherapy, art therapy, group excursions, occupational therapy, etc.)? - Supportive measures (including assistance with official matters)? |
| 2.2.5. | Do you have any wishes, suggestions, or ideas for other services that could be useful in a day hospice?  How do you think such services might make a difference for patients or for you as a relative? |
| 2.2.6. | In your personal opinion and experience, what general conditions or resources are needed for a day hospice to achieve the stated goals? |
| 2.2.7. | What kind of “human” resources do you think are needed for this? *(Prompt: Who should be on site, and what kinds of qualifications or specializations should they have? For example, physicians, nurses, pastoral caregivers, physical therapists, occupational therapists, or art therapists.)* |
| 2.2.8. | In your view, how should a day hospice be financed?  *(Prompt: What role should patients’ own contributions, public funding, health insurance, or donations play?)* |
| 2.2.9. | What kind of “organizational” resources are needed for this?  *(Prompt: For example, how should transportation to and from the day hospice be organized? Should patients come on their own, with relatives, by taxi, or by public transport? What operating hours would be ideal [full-day, half-day, or weekends]?)* |
| 2.2.10. | What kind of “social” resources do you think are needed to support day hospices?  *(Prompt: For example, how important are public awareness and social acceptance of serious illnesses, dying, and death? What role might volunteer work, donations, or general societal willingness to support, operate, and finance day hospices play? How do you see the involvement of politicians or health insurance companies?)* |
| 2.2.11. | How do you think the goals and services of a day hospice should be presented to the public?  *(Prompt: What could be good ways to make the purpose and offerings of a day hospice better known? Where and how should information be shared, e.g., through physicians, hospitals, the media, or community events?)* |
| 2.2.12. | Do you have any requests, suggestions, or ideas regarding additional resources that are required for day hospices achieve their goals? What difference would these resources make? |
| **2.3.** | **Personal relevance** |
| 2.3.1. | May I ask whether you personally feel that you benefit from your relative’s visits to the day hospice? |
| 2.3.2. | If so, what aspects in particular do you feel are most beneficial for you? |
| 2.3.3. | If not, could you tell me why you feel that way and what improvements you would like to see? |
| 2.3.4. | What is most important to you personally about the day hospice, and why? |
| 2.3.5. | Is there anything else you would especially like to see offered or included at the day hospice? |
| **2.4.** | **Naming** |
| 2.4.1. | We have now talked in detail about the topic of “day hospices.” To conclude, based on your experience to date, do you feel that the name “day hospice” is appropriate for such a facility?  *(Prompt: How does the name “day hospice” make you feel? Do you think it accurately reflects what the facility offers, or would another name seem more suitable?)* |
| 2.4.2. | Why yes or no? What alternative would you suggest, if any? |
| **2.5.** | **Completion** |
| 2.5.1. | That brings us to the end of our conversation. Is there anything else you would like to share with us regarding day hospice care? Is there anything we haven't discussed? Do you have any further wishes for the future?  *(Prompt: Is there anything we haven’t talked about that you feel is important? Do you have any additional thoughts or wishes for the future development of day hospices?)* |

**Table 2** Interview guide for patients

| **Question Number** | **Question** |
| --- | --- |
| **1.** | **Presentation** |
| 1.1. | To begin, could you tell me a little about yourself?  *(Prompt: For example, your age, family or marital situation, former or current occupation, and things that are important or enjoyable to you in everyday life.)* |
| 1.2. | Could you describe what a typical day looks like for you?  *(Prompt: What does your usual routine look like from morning to evening? Are there certain activities or moments in your day that are especially important or meaningful to you?)* |
| **2.** | **Day hospice: perception and evaluation** |
| **2.1.** | **Significance and impact of a day hospice** |
| 2.1.1. | Today, I would like to talk with you about the idea of a “day hospice.” Could you describe what you think is the main purpose and aim of a day hospice?  *(Prompt: What comes to mind when you think of a day hospice? What do you believe it is meant to offer or achieve for patients like yourself?)* |
| 2.1.2. | What immediate/direct effects do you personally hope to gain from attending a day hospice?  *(Prompt: Are there things you wish for yourself, for your relatives, or in general that you hope the day hospice can help with?)* |
| 2.1.3. | When you think a little further ahead (medium term), what effects or impact do you think a day hospice could have in the next few years?  *(Prompt: For example, what changes or positive effects do you expect for yourself, your relatives, for the healthcare system, or for society in general?)* |
| 2.1.4. | Looking further into the future, what long-term effects or impact (up to 10 years) do you think day hospices could have?  *(Prompt: For example, what difference could they make over the next several years for relatives, for the healthcare system, or for society as a whole?)* |
| **2.2.** | **Assessment of the importance of offers for achieving objectives** |
| 2.2.1. | In your personal view and experience, what services and facilities do you think are most important for a day hospice to offer in order to achieve the above-mentioned goals? |
| 2.2.2. | What kind of rooms or spaces do you think a day hospice needs in order to provide good care and support?  *(Prompt: For example, how important are areas such as quiet rooms, activity rooms, or outdoor spaces like a garden or balcony?)* |
| 2.2.3. | Which medical services do you think are most important for achieving the goals of a day hospice?  *(Prompt: e.g., services such as psychotherapy, wound care, pain management, and nutritional support)* |
| 2.2.4. | Which social or supportive services do you think are important in a day hospice?   - Cosmetic treatments (e.g., pedicures, haircuts, etc.)? - Complementary measures (e.g., aromatherapy, art therapy, group excursions, occupational therapy, etc.)? - Supportive measures (including assistance with official matters)? |
| 2.2.5. | Do you have any wishes, suggestions, or ideas for other services that might be useful in a day hospice? How do you think such services would make a difference? |
| 2.2.6. | In your personal opinion and experience, what general conditions or resources are needed for a day hospice to achieve its goals? |
| 2.2.7. | What kind of “human” resources do you think are needed in a day hospice to provide good care and support?  *(Prompt: Who should be on site and what kind of training or special skills should they have? For example, physicians, nurses, pastoral caregivers, physical therapists, occupational therapists, art therapists.)* |
| 2.2.8. | In your view, how should a day hospice be financed?  *(Prompt: What role should personal contributions, public funding, health insurance, or donations play? Do you think a mix of your own and external resources would be appropriate?)* |
| 2.2.9. | What kind of “organizational” resources do you think are needed for a day hospice to run smoothly?  *(Prompt: For example, how should transportation to and from the day hospice be arranged? Should patients come on their own, with family members, by taxi, or by public transport? What operating hours would you find most suitable [full-day, half-day, or weekends]?)* |
| 2.2.10. | What kind of “social” resources do you think are needed to support day hospices?  *(Prompt: For example, how important do you think public awareness and social acceptance of serious illnesses, dying, and death are? What role might volunteer work, donations, or the willingness of society, politicians, or health insurance providers to support and finance day hospices play?)* |
| 2.2.11. | How do you think the goals and services of a day hospice should be presented to the public?  *(Prompt: What would be good ways to make the purpose and offerings of a day hospice better known? Where and how should the information be shared?)* |
| 2.2.12. | Do you have any requests, suggestions, or ideas about additional resources that might be needed for a day hospice to achieve its goals? How do you think these additional resources could make a difference? |
| **2.3.** | **Personal relevance** |
| 2.3.1. | May I ask whether you personally feel that you benefit from visiting the day hospice? |
| 2.3.2. | If so, what aspects of a day hospice do you find particularly beneficial for yourself? |
| 2.3.3. | If not, could you tell me why you feel that way and what improvements you would like to see? |
| 2.3.4. | What is most important to you personally about the day hospice, and why? |
| 2.3.5. | Is there anything else you would like to see offered or included at the day hospice? |
| **2.4.** | **Naming** |
| 2.4.1. | We have now talked in detail about the topic of day hospices. To conclude, based on your experience so far, do you think the term “day hospice” is appropriate for such a facility? |
| 2.4.2. | Could you tell me why you feel that way? What alternative name would you suggest, if any? |
| **2.5.** | **Completion** |
| 2.5.1. | That brings us to the end of our conversation. Is there anything else you would like to share with us regarding day hospice care? Is there anything we haven't talked about that you think is important? Do you have any additional thoughts, ideas, or wishes for the future of day hospices? |
